# Associations between Allelic Variants of the Human IgH 3′ Regulatory Region 1 and the Immune Response to BNT162b2 mRNA Vaccine

**DOI:** 10.3390/vaccines9101207

**Published:** 2021-10-19

**Authors:** Mattia Colucci, Elisabetta De Santis, Beatrice Totti, Mattia Miroballo, Francesco Tamiro, Giovanni Rossi, Ada Piepoli, Gabriella De Vincentis, Antonio Greco, Alessandra Mangia, Rossella Cianci, Lazzaro Di Mauro, Giuseppe Miscio, Vincenzo Giambra

**Affiliations:** 1 Institute for Stem Cell Biology, Regenerative Medicine and Innovative Therapies (ISBReMIT), Fondazione IRCCS “Casa Sollievo della Sofferenza”, 71013 San Giovanni Rotondo, Italy; m.colucci@operapadrepio.it (M.C.); e.desantis@operapadrepio.it (E.D.S.); beatrice.totti9@gmail.com (B.T.); m.miroballo@css-mendel.it (M.M.); f.tamiro@operapadrepio.it (F.T.); 2 Department of Hematology and Stem Cell Transplant Unit, Fondazione IRCCS “Casa Sollievo della Sofferenza”, 71013 San Giovanni Rotondo, Italy; giovannirossi.fr@gmail.com; 3 Hospital Health Department, Fondazione IRCCS “Casa Sollievo della Sofferenza”, 71013 San Giovanni Rotondo, Italy; a.piepoli@operapadrepio.it (A.P.); g.devincentis@operapadrepio.it (G.D.V.); 4 Complex Structure of Geriatrics, Department of Medical Sciences, Fondazione IRCCS “Casa Sollievo della Sofferenza”, 71013 San Giovanni Rotondo, Italy; a.greco@operapadrepio.it; 5 Liver Unit, Department of Medical Sciences, Fondazione IRCCS “Casa Sollievo della Sofferenza”, 71013 San Giovanni Rotondo, Italy; a.mangia@operapadrepio.it; 6 Dipartimento di Medicina e Chirurgia Traslazionale, Università Cattolica del Sacro Cuore, Fondazione Policlinico Universitario “Agostino Gemelli”, IRCCS, 00168 Rome, Italy; rossellacianci@gmail.com; 7 Clinical Laboratory Analysis and Transfusional Medicine, Fondazione IRCCS “Casa Sollievo della Sofferenza”, 71013 San Giovanni Rotondo, Italy; l.dimauro@operapadrepio.it (L.D.M.); g.miscio@operapadrepio.it (G.M.)

**Keywords:** COVID-19, vaccine, HS1.2, IgH locus, antigen-specific B cells

## Abstract

The escalation of Coronavirus disease 2019 (COVID-19) has required the development of safe and effective vaccines against the severe acute respiratory syndrome coronavirus 2-associated (SARS-CoV-2), which is the causative agent of the disease. Here, we determined the levels of antibodies, antigen-specific B cells, against a recombinant GFP-tagged SARS-CoV-2 spike (S) protein and total T and NK cell subsets in subjects up to 20 days after the injection of the BNT162b2 (Pfizer–BioNTech) vaccine using a combined approach of serological and flow cytometry analyses. In former COVID-19 patients and highly responsive individuals, a significant increase of antibody production was detected, simultaneous with an expansion of antigen-specific B cell response and the total number of NK-T cells. Additionally, through a genetic screening of a specific polymorphic region internal to the 3’ regulatory region 1 (3’RR1) of human immunoglobulin constant-gene (IgH) locus, we identified different single-nucleotide polymorphic (SNP) variants associated with either highly or lowly responsive subjects. Taken together, these results suggest that favorable genetic backgrounds and immune profiles support the progression of an effective response to BNT162b2 vaccination.

## 1. Introduction

Coronavirus disease 2019 (COVID-19) is a new viral infection, caused by severe acute respiratory syndrome coronavirus 2-associated (SARS-CoV-2) [1] and declared a pandemic by the World Health Organization (WHO) in March 2020. Up to September 2021, COVID-19 has affected over 226,000,000 people and caused more than 4,600,000 deaths (WHO Coronavirus (COVID-19) Dashboard). Therefore, the development of safe and effective vaccines against SARS-CoV-2 has been urgently needed [2,3,4].

The BNT162b2 (Pfizer–BioNTech) RNA-based vaccine was the first to obtain a conditional marketing authorization by the European Medicines Agency (EMA) for COVID-19 prevention [5]. This consists of nucleoside-modified mRNA encapsulated in lipid nanoparticles, encoding the SARS-CoV-2 full-length spike (S) with two proline mutations to block pre- and post-fusion conformations [2]. The receptor-binding domain (RBD) of SARS-CoV-2 spike (S) protein plays an important role in the viral infection process, fostering the binding of the virus to host cells [6]. This is the main target of the cellular and humoral immune response inducing the production of antigen-specific antibodies [7]. Moreover, recent studies have reported that a single dose of BNT162b2 vaccine induces anti-SARS-CoV-2 neutralizing antibodies with a highly detectable titer [3,8,9,10,11] and expands specific T cell components, including T_H_1 type CD4+ and IFNg+ CD8+ T cells [5,12,13,14]. Taken together, these results support the efficacy of the BNT162b2 vaccine against SARS-CoV-2 and suggest that pre-existing innate and adaptive immune conditions might enforce the response to the vaccination.

Genetic differences and environmental factors can contribute to an altered immune-response to different vaccines [15]. In the last few years, key genetic determinants of vaccine-induced immune response have been reported [16,17]. The 3’ regulatory region 1 (3’RR1) of the human immunoglobulin heavy chain (IgH) locus has a relevant role in the immune response, being involved in the B-cell maturation, Ig class switch and expression [18,19]. In the human IgH locus, several polymorphic regions have also been described as functional to the activity of 3′RR [20,21] and associated with different human immune disorders [22,23,24,25,26].

The objectives of this study were to determine the levels of antibodies, antigen-specific B cells against a recombinant GFP tagged SARS-CoV-2 S protein and total T and NK cell subsets in subjects up to 20 days after the injection of the BNT162b2 (Pfizer–BioNTech) vaccine, using a combined approach of serological and flow cytometry analyses. Additionally, we performed a genetic screening of a specific polymorphic region internal to the 3’ RR1 of human IgH locus to highlight key genetic determinants of vaccine-induced immune response and favorable genetic backgrounds that might enforce the immune response to vaccination against SARS-CoV-2.

## 2. Materials and Methods

Human samples and count blood cells. Blood samples were collected from 160 healthcare workers at the Casa Sollievo della Sofferenza Hospital in Southern Italy. All participants were Caucasian people from the same geographic area, and 52% of them were female. The median age was 50 years, and 23% of individuals (*N* = 37) were former COVID-19 patients (ex-COVID-19), completely negative to SARS-CoV-2 molecular testing for at least for two months at the time (day 0) of BNT162b2 mRNA vaccine administration. Among the seronegative participants at day 0 (naïve), we distinguished low (*N* = 30) and high (*N* = 93) responsive individuals based on the lower 10th percentile of anti-SARS-CoV-2 IgG sampling distribution, which set the cut-off point at 3.05 AU/mL, as determined by the SARS-CoV-2 IgG ADVIA Centaur immunological assay (Siemens Healthineers). Blood samples were collected from 57 unvaccinated healthy donors as control (Table 1). All samples were collected after appropriate institutional approvals (Casa Sollievo della Sofferenza Research Ethics Boards, IRB code: GEN-COVID—V1_04 Mar 21), and signed informed consent was attained under guidelines established by the Declaration of Helsinki. The complete blood count was performed at the Casa Sollievo della Sofferenza Hospital with the Sysmex-XT-4000i automated hematology analyzer, following standard procedures.

SARS-CoV-2 IgG Assay. The Siemens Healthineers SARS-CoV-2-IgG (COV2G) assay was employed for qualitative and semi-quantitative detection of IgG antibodies against SARS-CoV-2 in blood serum using the Atellica-IM-Analyzer and following the manufacturer’s procedures. Antibody levels were measured before vaccination for control samples and at day 20 after dose 1. Seropositive status due to prior SARS-CoV-2 exposure was determined on a previously established cutoff [27].

Cell culture and transfection. HEK-293T cells were cultured in DMEM medium supplemented with 10% fetal bovine serum (FBS), 1 mM sodium pyruvate, 2 mM L-glutamine, 100 units/mL penicillin and 100 μg/mL streptomycin (ThermoFisher, Waltham, MA, USA). The pcDNA3-SARS-CoV-2-S-RBD-sfGFP plasmid encoding the Receptor Binding Domain (RBD) of SARS-CoV-2 protein S (spike) fused with sfGFP fluorescent marker was derived from Addgene (cod. #141184) [6]. An empty vector, expressing only the sfGFP, was used as control. HEK-293T cells were transiently transfected using Polyethylenimine Linear, MW25000, Transfection Grade (PEI 25K) (Polysciences, Inc., Warrington, PA, USA), as described previously [28]. After transfection, HEK-293T cells were cultured for 2 days and subsequently assessed for GFP expression by flow cytometry before protein purification.

Protein purification and Western blot assay. HEK-293T cells, transiently transfected with pcDNA3-SARS-CoV-2-S-RBD-sfGFP plasmid or control were collected, washed in ice-cold phosphate-buffered saline and then lysed in ice-cold 50 mM Tris-HCl (pH 7.4), 1% Nonidet P-40, 0.25% sodium deoxycholate, 150 mM sodium chloride, 1mM sodium orthovanadate, 1mM sodium fluoride, 2.5 mM sodium pyrophosphate, 1 mM EDTA, 1 mM phenylmethylsulphonyl fluoride, and protease inhibitor cocktail (cat #539134, Calbiochem Merck KGaA, Darmstadt, Germany). Whole cell lysates were incubated at 95 °C for 10 min, loaded on SDS-PAGE gels and then transferred to Hybond-ECL membranes (Amersham Biosciences Corp, Amersham, UK). The membranes were blocked with 5% milk/0.3% TBS-Tween20 at 4 °C for 1 h and then probed with primary antibodies against GFP (1:1000 dilution; cat.ab290, AbCam, Cambridge, UK) or β-Actin (1:6000 dilution; cat.A1978, Sigma-Aldrich, Darmstadt, Germany). HRP-conjugated secondary antibodies (Cat. NEF812001EA, Perkin Elmer, Waltham, MA, USA) were used at 1:10,000 dilution. The chemiluminescent signal was detected with enhanced chemiluminescence (ECL) (cat.32106, Pierce, ThermoFisher, Waltham, MA, USA) and subsequently autoradiography.

Flow Cytometry. A total of 30 μL of fresh blood from control or vaccinated individuals was treated with 300 μL of ammonium chloride solution (Cat. #07850, Stemcell Technologies, Vancouver, BC, Canada). After red blood cell (RBC) lysis, cells were washed with DPBS and resuspended in 200 μL of DPBS with 35 μg of protein extracts, containing the recombinant sfGFP-tagged SARS-CoV-2 S/RBD protein or sfGFP only as control. Subsequently, cells were washed with DPBS and then stained for surface markers (Table S1) in DPBS with BD Horizon Brilliant Stain Buffer (Becton Dickinson, Franklin Lakes, NJ, USA) for 20 min at room temperature. The DRAQ7 fluorescent DNA dye (1:1000 dilution; cat. D15106, ThermoFisher, Waltham, MA, USA) was used to identify alive cells. We performed FACS assays on FACS Calibur, Canto2 (Becton Dickinson, Franklin Lakes, NJ, USA) and MoFlo Astrios cell sorter (Beckman Coulter) and used FlowJo software (Becton Dickinson) for analysis and the visualization of multiparameter data through the tSNE algorithm [29].

Purification of human genomic DNA. DNA purification from whole blood was carried out according to the protocol from the QIAamp DNA Mini and Blood Mini kit (QIAGEN). A total of 200 uL of 192 blood samples were thawed and incubated with proteinase K and lysis buffer at 56 °C for 10 min to protect the DNA from nuclease digestion. This mixture was applied to the QIAamp spin column, and DNA was bound to the membrane, purified and eluted from the membrane. The analysis of the DNA concentration and purity was performed with ThermoScientific NanoDrop one microvolume UV-Vis spectrophotometer.

Amplification and sequencing of allelic variants of the human IgH 3′ RR1. A long PCR assay was initially performed on the selected genomes, amplifying a 5.4 Kb region of IgH3′EC-1 that includes both hs1.2 and hs3 enhancers. The large genomic fragment was amplified by the primer SA2.5 (5′-GGATCCCTGTTCCTGATCACT G-3′) and A2R (5′-GCCCTTCCTGCCAACCTG-3′) using the Expanded Long Template PCR System (Roche). The optimal reaction conditions were: 3.75 units of Taq DNA polymerase, Expanded Long Template buffer 1 (10X conc. with 17.5 mM MgCl_2_), dNTP mix (350 μM), primers (300 nM), DNA (100 ng), Betaine (0,9 M) and water for a final volume of 50 μL. The sample reactions were placed in the thermal block cycler and performed at 94 °C for 2 min, followed by 10 cycles at 94 °C for 10 s, 64 °C for 30 s and 68 °C for 4 min, followed by 30 cycles at 94 °C for 15s, 64 °C for 30 s, 68 °C for 4 min plus 20 s cycle elongation for each successive cycle and one final extension at 68 °C for 7 min. The PCR products were checked using 1% agarose gel.

At the following, four different Nested PCRs were done in order to efficiently amplify the polymorphic regions of IgH3′EC-1 (Table S2). Nested PCR1 was carried out using two primers, P3FFrw (5′-GACTCATTCTGGGCAGACTTG-3′) and D3Rev (5′-GTCCTGGTCCCAAAGATGG -3′). The amplification was performed in a PCR tube containing 10X PCR buffer (minus Mg), 50 mM MgCl_2_ (1.5 mM final conc.), 0.2 mM dNTP mix, 0.5 μM of each of P3FFrw and D3Rev primers, 1/10 of the volume of the Long PCR reaction and 1.0–2.5 U Taq DNA polymerase recombinant, Invitrogen. The final volume of the reaction mixture was topped up to 50 μL with sterile distilled water. The optimized thermocycler conditions for the reaction were initial denaturation at 94 °C for 3 min, 30 cycles at 94 °C for 45 s, 55 °C for 30 s, 72 °C for 33 s and a final extension at 72 °C for 10 min. The second, third and fourth Nested PCR amplification were carried out using same final concentration of the reagents as described above, except replacing the primers Nested2Frw (5′-TCTCCTGTTCTCTGACCATC-3′), Nested2Rev (5′-GTCACATCCATACCCACACT-3′), Nested3Frw (5′-TCCCTGTCCCTGTCTCTTAT-3′), Nested3Rev (5′-AATACCCAAAATAGCCCTGT-3′), Nested4Frw (5′-ATTCAAGAGGCTTCAGGAGA-3′) and Nested4Rev (5′-TTCCTTTTCTGAGCATGTATC -3′). The thermocycler conditions were also kept the same except for the time of elongation: 40s for Nested PCR2 and 36 s for Nested PCR3/4. The PCR products were controlled using 1.5% agarose gel and sequenced by the Sanger sequencing service of Eurofins Genomics. The allelic variants of each SNP were determined using the DNA analyzing program, 4Peaks (http://nucleobytes.com/index.php/4peaks, accessed on 21 July 2021).

Statistics. We used GraphPad-Prism 8.4.3 software for the visualization and statistical analyses of quantitative data, including Pearson and Spearman correlations and Welch’s *t*-test. The chi-square and *p*-values of deviations from the Hardy–Weinberg equilibrium were determined using the publicly available software at the link http://www.husdyr.kvl.dk/htm/kc/popgen/genetik/applets/kitest.htm (accessed on 21 July 2021).

## 3. Results

Determination of antibody levels against SARS-CoV-2.

It has been recently reported that SARS-CoV-2-specific antibodies are detectable in the majority of seronegative individuals at day 20 after the first dose of the BNT162b2 mRNA vaccine [30,31]. To assess the serological level of anti-SARS-CoV-2 spike (S) IgG antibody in our sample cohort, we performed an S-binding chemiluminescent immunoassay [32]. We determined the antigen-specific antibody response to the vaccine for all participants at day 20 and distinguished the low-response individuals in the lower 10th percentile of antibody titer distributions (Table 1). Of interest, the levels of anti-SARS-CoV-2 spike-specific IgG were significantly increased in individuals previously exposed to SARS-CoV-2, as recently reported [8,33] (Figure S1A). We also found a significant slight reduction in people older than 60 years of age (*p*-value = 0.010, two-tailed Mann–Whitney test) (Figure S1B) and no difference between male and female at the considered time point (*p*-value > 0.05, two-tailed Mann–Whitney test) (Figure S1C). Nonetheless, an increase of high-response individuals was detected in the female cohort compared to male subjects (52 female vs. 41 male), suggesting that sex may influence the antibody titer too.

Immunophenotypic characterization of peripheral blood mononuclear cells (PBMCs)

**Figure 1:**
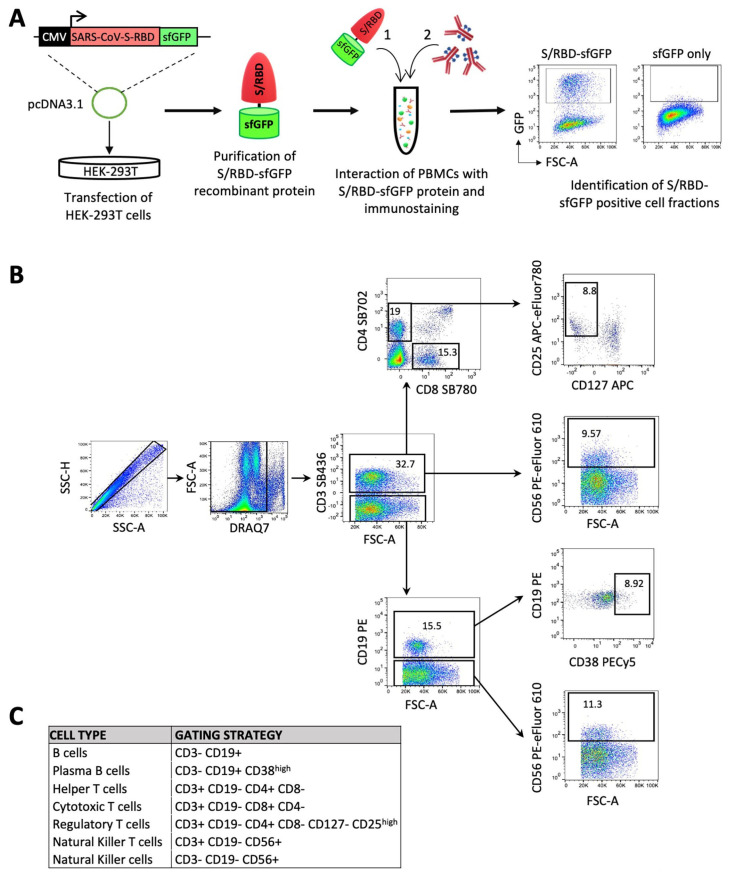
Immunophenotypic characterization of peripheral blood mononuclear cells (PBMCs) in response to BNT162b2 vaccine. (**A**) Schematic overview of an experimental approach for the identification of S/RBD-specific B cells and total T and NK cell subsets. Specifically, HEK-293T cells were transiently transfected with pcDNA3.1-SARS-CoV-2-S-RBD-sfGFP vector, encoding the receptor binding domain (RBD) of the SARS-CoV-2 protein S (spike) fused with sfGFP fluorescent marker. The recombinant GFP-tagged S/RBD protein was purified after 2 days from transfection and employed for the immunostaining of mononuclear cells (PBMCs) after red blood cell (RBC) lysis. Purified cells were firstly exposed to the recombinant sfGFP-tagged SARS-CoV-2 S/RBD protein or only sfGFP as control. Subsequent to washing, cells were labeled with a panel of 8 fluorophore-conjugated antibodies against lineage-specific cell surface markers in order to identify the B, T and NK cell subsets and GFP positive fraction in B cell populations.(**B**) Gating strategy for classifying the different subpopulations of lymphocytes with the reported panel (Table S1). Fluorescence minus one (FMO) controls were used to set up all gates. Singlets were initially discriminated on SSC-H and SSC-A, followed by the exclusion of non-viable cells with DRAQ7 far-red fluorescent DNA dye. Subsequent gating identified CD3+ cells, followed by the identification of CD4+CD8− helper T cells and CD4−CD8+ cytotoxic T cells. Within CD4+ cell fraction, regulatory T cells were discriminated as CD127− CD25^high^ cells. Natural killer T-cells were also identified as CD56+ cells in the CD3+ fraction. B cells were identified as CD3−CD19+, and plasma B cells were differentiated as CD38^high^ within the subset of B cells. Finally, NK cells were discriminated as CD3− CD56+ CD19−. (**C**) Table summarizing the gating strategy described in B.

**Figure 2:**
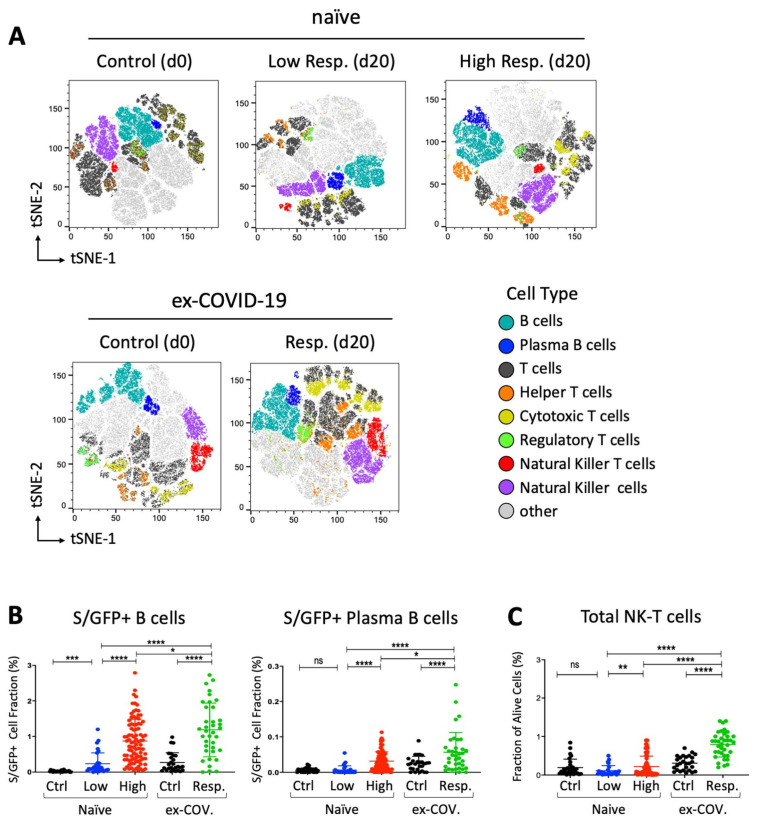
Distribution of spike/RBD-specific B cells and total NK-T cell subset in response to BNT162b2 vaccine. (**A**) tSNE map based on the flow cytometry data from participants, subdivided into low- (*N* = 30) and high (*N* = 93)-response “naïve” individuals at day 20 and control (*N* = 30) at day 0. Flow cytometry data from seropositive (ex-COVID-19) individuals at days 0 and 20 were also analyzed and reported as response (*N* = 37) and control (*N* = 27) respectively. Only individuals from the same geographic area and of similar ages have been collected and selected for the reported assay. A total of 100,000 live lymphocytes for each sample were considered for concatenation and downsampling. t-SNE dimensional reduction was performed for the visualization of cell subsets for each cohort in two dimensional plots using standard parameters (perplexity = 30, theta = 0.5). (**B**,**C**) GFP+ B cell fraction as result of interaction with the recombinant GFP-tagged SARS-CoV-2 spike RBD protein (S/GFP+) and alive cell fraction of the indicated NK-T cell subset. Seronegative participants at day 0 are reported as “naïve” and subdivided in low- (in blue, *N* = 30) and high (in red, *N* = 93)-response individuals based on the lower 10th percentile of anti-SARS-CoV-2 IgG sampling distribution. Former COVID-19 patients at day 20 after vaccination are indicated as “ex-COV” (in green, *N* = 37). The S/RBD-specific cellular response of unvaccinated ex-COVID (*N* = 27) and naïve (*N* = 28) samples are reported in black as control. Welch’s *t*-test statistical analysis was performed on the percentage values of different cell fractions. ns, not significant; *, *p* < 0.05; **, *p* < 0.01; ***, *p* < 0.001, **** *p* < 0.0001 (two-tailed unpaired Welch’s *t*-test).

To assess the immunophenotypic cellular changes up to day 20 after the first dose of the vaccine, we designed a multiparameter flow cytometry panel, including fluorophore-conjugated antibodies against surface lineage markers for the detection of discrete subsets of B, T and natural killer (NK) cells. Specifically, in this flow cytometry assay, the peripheral blood mononuclear cells (PBMCs) of all participants were incubated with protein extracts that included the recombinant protein constituted by the receptor binding domain (RBD) of the SARS-CoV-2 S glycoprotein, fused to the superfolder green fluorescent protein (sfGFP) [6], in order to identify the S/RBD-binding B cells (Figure 1). Initially, t-SNE dimensional reduction [29] was performed for visualization of cell subsets in two dimensional plots using standard parameters (perplexity = 30, theta = 0.5). In this assay, the samples were divided in two main groups depending whether they were originated from seronegative (naïve) or seropositive (ex-COVID-19) participants at the time (d0) of injection of the first dose of BNT162b2 vaccine. The “naïve” subjects were further subdivided into low- and high-response individuals, based on the level of antibody titer at day 20 (Figure S1A). Samples derived from unvaccinated seronegative (naïve) or seropositive (ex-COVID-19) people were also analyzed and included as control. We observed that the proportions of all major lineages, such as total lymphocytes, B and T cells, were highly similar in all participants (Figure 2A) and confirmed these observations by complete blood cell counts, including neutrophiles, monocytes, eosinophiles and basophiles (Figure S2).

We also determined the fraction of GFP-positive B cells binding the recombinant GFP-tagged S/RBD protein. As expected, we observed an increase of total and plasma B cells, interacting with S/RBD protein in all subjects at day 20 after vaccination. Of interest, the level of B cell subsets was higher in seropositive (ex-COVID-19) individuals with respect to the other cohorts (Figure 2B). Interestingly, we also found a statistically significant enrichment of total NK-T cells after exposure to the BNT162b2 vaccine. Notably, the increase of NK-T cells was higher in former COVID-19 patients than highly responsive seronegative subjects (*p*-value < 0.0001, two-tailed Welch’s *t*-test) (Figure 2C). Taken together, these data suggest that the efficacy of the immune response to the vaccine involves cellular changes that might be enforced by pre-existing immunological states in former COVID-19 patients.

**Figure 3:**
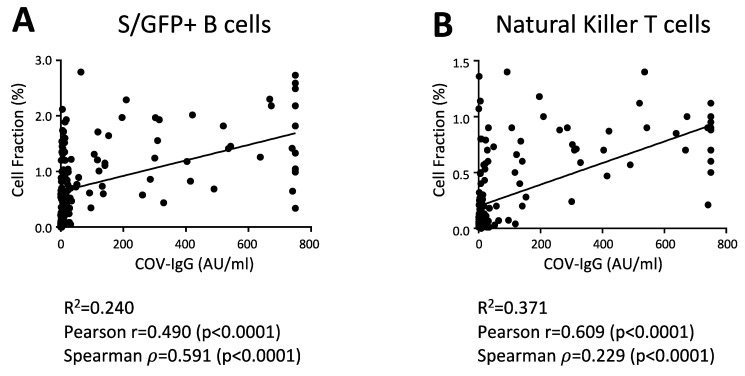
The levels of anti-SARS-CoV-2 IgG directly correlate with the S/RBD-binding B cells and total NK-T cells. Correlation analysis between the anti-SARS-CoV-2 IgG levels and the abundance of indicated cell populations, including B cells, interacting with the SARS-CoV-2 S RBD protein (S/GFP+ cells) (**A**) and total NK-T cells (**B**) in all vaccinated participants (*N* = 160). Pearson correlation coefficient (r), Spearman’s rank correlation coefficient (ρ) and their statistical significance (*p*-value) are reported in the graphs. Linear correlation was evaluated through the linear regression model. The linear regression line in black and R squared (R^2^) are also shown in the graphs.

To assess any direct correlation between antibody levels and cellular changes, we determined the Pearson (r) and Spearman (ρ) correlation coefficients between S/RBD-interacting B cells and anti-SARS-CoV-2 IgG levels at day 20 after vaccine. As expected, the antibody titer was directly correlated with the levels of GFP+ B cells in the considered cohort (Figure 3A). Of interest, total NK-T cells were also significantly correlated with the anti-SARS-CoV-2 IgG level (Figure 3B), suggesting that these cell subsets could enhance the antibody production and B cell response to the BNT162b2 vaccine, as reported previously [34,35]. Finally, we did not found correlations between anti-SARS-CoV-2 IgG levels and other cell subsets, including total NK and T cell subsets.

Characterization of allelic variants in the human IgH 3’ RR1.

**Figure 4:**
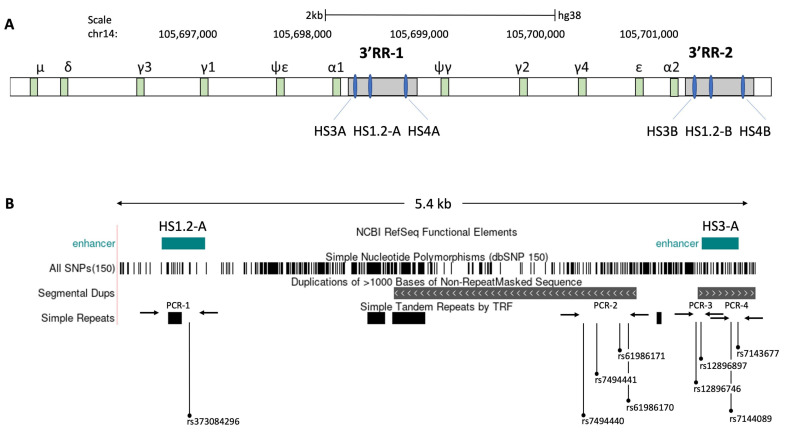
Schematic map and positions of the detected SNPs in the 3’ regulatory region 1 (3’RR1) of the human immunoglobulin heavy chain (IgH) locus. (**A**) The Ig heavy-chain region on human chromosome 14 is characterized by two duplicated regulatory regions (3′ RR), located at 3′ of immunoglobulin (Ig) heavy chain genes. Each 3′ RR locus contain 3 enhancers (HS3, HS1.2 and HS3). (**B**) Schematic map of 5.4 kb region amplified by the long PCR (see method) for the detection of considered SNPs, which also includes the HS3A and HS1.2-A enhancers and regions with simple tandem repeats. After the amplification of long PCR, four different nested PCRs, highlighted by the arrows, were subsequently performed in order to identify 9 SNPs (rs373084296, rs7494440, rs7494441, rs61986170, rs61986171, rs12896746, rs12896897, rs7144089 and rs7143677) by Sanger DNA sequencing.

The fast expansion of high throughput sequencing has underlined the genetic differences, as well as identified several polymorphisms in genes, impacting the immune responses to vaccines, including hepatitis B and smallpox [15,36,37]. To highlight the different genetic backgrounds between high- and low-response individuals to the BNT162b2 vaccine, we sequenced four specific polymorphic regions of the 3’ regulatory region 1 (3’RR1) of the human immunoglobulin heavy chain (IgH) locus [24,38] in the vaccinated seronegative (naïve) subjects (Figure 4). Specifically, the allelic frequencies of 9 single-nucleotide polymorphisms (SNPs), situated on the palindrome region downstream of HS1.2 enhancer in the 3′RR1 of IgH locus were assessed in relationship to the different antibody levels of seronegative (naïve) subjects after vaccination (Table 2). Interestingly, significant differences in the genotype distribution were found in 5 of the 9 SNPs (rs373084296, rs7494441, rs12896746, rs12896897, rs7144089) in subjects with high levels of antibodies (*p* value < 0.01 for rs12896746, and *p* value < 0.001 for the others). While in subjects with low levels of antibodies, we have found significant differences only in 3/9 SNPs (rs12896746, rs12896897, rs7144089) (*p* value < 0.01). The Table 2 summarizes the chi-square and *p*-value of deviations from Hardy–Weinberg equilibrium related to the expected genotypes and observed genotypes for each SNP in both sample cohorts. Specifically, we observed an increase of homozygosity for adenine (A) in the rs373084296 and rs12896746 polymorphisms in individuals with a lower antibody level in response to the vaccine. At the contrary, highly responsive individuals were enriched with homozygosity for guanine (G) in both SNPs (Table 3). Furthermore, high levels of antibodies were linked to the presence of homozygosity for thymine (T) in rs7494441 and rs12896897 SNPs, while low levels of antibodies correlated to heterozygosity of cytosine-thymine (CT) (Table 3). For the rs7144089, the homozygosity for C were also linked to higher levels of antibodies in respect to homozygosity of G and heterozygosity of CG. The other genotypes were in Hardy–Weinberg equilibrium and did not show statistically significant differences considering the different antibody levels (Table 3). Taken together, these findings highlight the relevant role of 3’RR1 in the human IgH expression and suggest a possible genetic predisposition to develop a more efficient response to BNT162b2 vaccine.

## 4. Discussion

Emerging studies including the serological level of SARS-CoV-2-specific antibodies in response to the BNT162b2 (Pfizer–BioNTech) mRNA vaccine have suggested that individuals previously infected with SARS-CoV-2 have naturally acquired an immunity and thus, proposed alternative approaches for the vaccination of former COVID-19 patients [8,30,31,33]. In order to explore the pre-existing and different immune profiles after anti-SARS-CoV-2 vaccination in lowly versus highly responsive individuals, as well as subjects previously infected with SARS-CoV-2, we analyzed blood samples, collected from 160 healthcare workers at a large medical center in Southern Italy up to 20 days after the injection of the BNT162b2 mRNA vaccine; 23% of subjects were former COVID-19 patients, completely negative to SARS-CoV-2 molecular testing for at least for two months. Previously described clinical trials reported that the BNT162b2 mRNA vaccine is safe and 95% effective against COVID-19 [3,39]. Despite not being designed to assess the efficacy of a single-dose regimen, a divergence between placebo and vaccine recipients starts at 12 days after the first dose, an indication of an early onset of a partially protective immunization, with an efficacy of 52% in the interval between the first and second dose [3,12,40]. In this work, we aimed to understand the immunological landscape and response to the BNT162b2 mRNA vaccine, focusing on the antigen-specific immune profile against SARS-CoV-2 S and genetic variations. In line with previous reports [41,42,43,44], we observed that mRNA vaccines are potent drivers of B cell immune response against the SARS-CoV-2 spike protein in both naïve and pre-infected individuals, but despite several similarities, the latter displayed a higher antigen-binding capacity after a single vaccine dose.

In addition to the expected increase in total and plasma B cells interacting with the antigen, we noted that ex-COVID-19 participants also had a greater level of total NK-T cells with respect to the highly responsive group, suggesting that this cell subset might enforce the response to BNT162b2 vaccine. Surprisingly, a high level of total NK-T cells in ex-COVID-19 participants was also detected before the injection of the first dose. Overall, our data suggest that there are changes in the cellular immunological landscape between participants that were exposed to SARS-CoV-2 infection and naïve participants, mainly characterized by an increased response in the previously exposed group. Moreover, differences in naïve high and low participants’ immune response also point towards a different pre-existing immunological landscape that correlates with a positive response to the vaccine.

In general, the use of the recombinant spike protein for the selection of interacting B cells have been successfully employed, particularly in those studies that aimed to characterize new epitopes of spike protein and to synthesize increasingly updated monoclonal antibodies. In their work, Xie et al. generated Sars-CoV-2 specific antibodies starting from the isolation of B cells of infected individuals and testing them for their specificity in antigen-binding to Sars-CoV-2 RDB subunit [45]. The sequencing of RDB-interacting B cells and the subsequent cloning of VH and VL sequence have led to the production of monoclonal anti-RDB antibodies that displayed new binding sites that are different from other known antibodies. Our cytofluorimetric assay can be a quick screening method for antigen-interacting B cells, potentially facilitating the development of recombinant antibodies together with various mutations that could arise over the global infected individuals along with COVID- 19 evolution.

In this study, we have also reported an association between the allelic variants and genotypes of polymorphic DNA region of the 3′ regulatory region 1 in the human IgH locus with different antibody levels in response to BNT162b2 vaccine. The observed changes might be in part due to the DNA taq polymerase (Invitrogen), used in the nested PCR, which does not have proofreading activity and has an error rate of 2.2 × 10^−5^ errors per nt per cycle. Nonetheless, in the considered SNPs of human 3′RR1, we observed mainly alterations in guanine or cytosine nucleotides as reference or alternative variants. This suggests the DNA methylation status of these sites might diverge among individuals and directly affect the DNA binding of regulatory protein factors or the three-dimensional conformation of the locus during B cell maturation, V(D)J recombination and Ig expression, as previously reported in mice [46,47]. Taking together, these results support the idea that a possible genetic predisposition fosters a favorable immune response and that a personalized approach might be needed for lowly or nonresponsive individuals to vaccination against SARS-CoV-2. Future studies could help in elucidating the underlying causes of these different responses that could arise from a history of previous similar infections, or genetic, individual, and environmental differences.

## 5. Conclusions

In this study, we determined the levels of antibodies and antigen-specific B cells against a recombinant GFP tagged SARS-CoV-2 spike (S) protein in subjects up to 20 days after injection with the BNT162b2 (Pfizer–BioNTech) vaccine using a combined approach of serological and flow cytometry analyses. Of interest, we found that the antigen-specific B cell response and the total number of NK-T cells directly correlate with the anti-SARS-CoV-2 IgG level, suggesting that specific immune cell subsets might support an effective response to BNT162b2 vaccination. Additionally, through a genetic screening of single-nucleotide polymorphic (SNP) variants in the human IgH locus, we have also highlighted favorable genetic backgrounds in highly responsive subjects to COVID-19 vaccination. Taken together, our findings support the idea that genetic restrictions and/or adverse immunological status may restrain an effective antibody production against the SARS-CoV-2 virus and alter the protective immune responses to vaccines. This may be significantly relevant in light of the rapid development of SARS-CoV-2 variants that will require constant adjustments in antibody-production procedures.

## Figures and Tables

**Table 1:** Demographic distribution and anti-SARS-CoV-2 IgG plasma levels of participants. Whole blood samples were collected from 160 healthcare workers at a large medical center in Southern Italy. All participants are Caucasian people from the same geographic area, and 52% of them were female. The median age was 50 years, and 23% of individuals (*N* = 37) were former COVID-19 patients (ex-COVID-19), completely negative to SARS-CoV-2 molecular testing for at least for two months at the time (day 0) of BNT162b2 mRNA vaccine administration. Among the seronegative participants at day 0 (naïve), we distinguished low (*N* = 30) and high (*N* = 93) responsive individuals based on the lower 10th percentile of anti-SARS-CoV-2 IgG sampling distribution, which set the cut-off point at 3.05 AU/mL, as determined by the SARS-CoV-2 IgG ADVIA Centaur immunological assay (Siemens Healthineers).

		Demographic and Clinical Characteristics of Partecipants									
		Sampling Distribution						COV-IgG			
		Counts	% of Tot.	M	F	Low Responsive (COV-IgG < 3.05 AU/mL)	High Responsive (COV-IgG ≥ 3.05 AU/mL)	Min (AU/mL)	Max (AU/mL)	Mean	SD
Individuals	Naive	123	76.9%	57	66	30	93	0	>750	77.8	196.6
	ex-COVID-19	37	23.1%	20	17	2	35	0.59	>750	306.9	275.6
	Tot.	160	100%	77	83	30	93	0	>750	130.8	237.2
		Counts	% of tot.	Average age	ex-COV.(%)	Low Responsive (COV-IgG < 3.05 AU/mL)	High Responsive (COV-IgG ≥ 3.05 AU/mL)	Min (AU/mL)	Max (AU/mL)	Mean	SD
Gender	M	77	48.1%	50	26%	16	41	0	>750	140.6	240.5
	F	83	51.9%	49	20.5%	14	52	0	>750	121.6	235
		Counts	% of tot.	Gender	ex-COV.(%)	Low Responsive (COV-IgG < 3.05 AU/mL)	High Responsive (COV-IgG ≥ 3.05 AU/mL)	Min (AU/mL)	Max (AU/mL)	Mean	SD
Age Class	21–30	16	10%	9M–7F	25%	1	11	1.73	>750	126.84	217.9
	31–40	22	13.7%	8M–14F	27.3%	3	13	0.28	>750	136.36	251.9
	41–50	34	21.3%	18M–16F	20.6%	5	22	0.53	673	136.98	238.2
	51–60	52	32.5%	20M–32F	21.2%	10	31	0	>750	125.52	238
	60+	36	22.5%	22M–14F	25%	11	16	0	>750	130.83	246.9

**Table 2:** Allele frequencies and distribution of detected 9 SNPs in seronegative individuals, lowly and highly responsive to the BNT162b2 vaccine. In the left side, the allele frequencies of detected 9 SNPs (rs373084296, rs7494440, rs7494441, rs61986170, rs61986171, rs12896746, rs12896897, rs7144089 and rs7143677) associated with the individuals with low and high antibody levels in response to the BNT162b2 vaccine are stated. In the right side, the chi-square (χ^2^) is reported, and P values are determined for the deviation from Hardy–Weinberg equilibrium in each cohort of individuals, as in Table 2A. Nt, nucleotide; ns, not significant; **, *p* < 0.01; ***, *p* < 0.001.

SNP Code	Nt.	Low	High		Low	High
rs73084296	A	0.74	0.54	χ^2^	0.10	6.37
	G	0.26	0.46	*p*-value	NS	**
rs7494440	C	0.64	0.28	χ^2^	2.33	3.30
	G	0.36	0.72	*p*-value	NS	NS
rs7494441	C	0.66	0.54	χ^2^	3.54	25.77
	T	0.34	0.46	*p*-value	NS	***
rs61986170	G	0.80	0.77	χ^2^	0.00	1.12
	A	0.19	0.22	*p*-value	NS	NS
rs61986171	C	0.82	0.75	χ^2^	1.14	0.24
	T	0.71	0.25	*p*-value	NS	NS
rs12896746	A	0.38	0.32	χ^2^	5.56	11.49
	G	0.61	0.67	*p*-value	**	***
rs12896897	C	0.55	0.53	χ^2^	5.37	24.33
	A	0.44	0.46	*p*-value	**	***
rs7144089	C	0.46	0.51	χ^2^	7.26	23.37
	G	0.54	0.48	*p*-value	**	***
rs7143677	A	0.72	0.71	χ^2^	3.31	2.68
	G	0.27	0.28	*p*-value	NS	NS

**Table 3:** Distribution of observed (Obs.) and corresponding expected (Exp.) genotypes under Hardy–Weinberg equilibrium in both cohorts of seronegative individuals with low and high antibody levels in response to the BNT162b2 vaccine. Only the SNPs with populations in Hardy–Weinberg disequilibrium are reported. The difference between low vs. highly responsive subjects for the observed genotype in each SNP is indicated as “Δ(Low-High)”.

**rs73084296**						**rs7494441**					
	**Low**		**High**				**Low**		**High**		
**Genotype**	Obs. (%)	Exp. (%)	Obs. (%)	Exp. (%)	Δ(Low-High)	Genotype	Obs. (%)	Exp. (%)	Obs. (%)	Exp. (%)	Δ(Low-High)
A/A	56	54.8	21.2	28.9	34.8	C/C	52	43.6	44.1	28.8	7.9
A/G	36	38.5	65.2	49.7	−29.2	C/T	28	44.9	19.1	49.7	8.9
G/G	8	6.8	13.6	38.5	−5.6	T/T	20	11.6	36.8	21.5	−16.8
**rs12896746**						**rs12896897**					
	**Low**		**High**				**Low**		**High**		
**Genotype**	Obs. (%)	Exp. (%)	Obs. (%)	Exp. (%)	Δ(Low-High)	Genotype	Obs. (%)	Exp. (%)	Obs. (%)	Exp. (%)	Δ(Low-High)
A/A	3.8	14.8	1.5	10.5	2.3	C/C	43.1	28.6	42.3	31.1	−0.8
A/G	69.2	47.3	61.8	43.8	7.4	C/T	20.8	49.8	26.9	49.3	6.1
G/G	26.9	37.9	36.8	45.8	−9.9	T/T	36.1	21.6	30.8	19.6	−5.3
**rs7144089**											
	**Low**					**High**					
**Genotype**	Obs. (%)		Exp. (%)			Obs. (%)			Exp. (%)		Δ(Low-High)
C/C	34.8		20.8			41.5			26.6		−6.7
C/G	21.7		49.6			20			50		1.7
G/G	43.5		29.5			38.5			23.5		5

## Supplementary Materials

The following are available online at https://www.mdpi.com/article/10.3390/vaccines9101207/s1, Figure S1: anti-SARS-CoV-2 IgG level induced at day 20 by BNT162b2 vaccine, Figure S2: Distribution of blood cell counts in response to BNT162b2 vaccine at day 20. Table S1: Panel of cell surface markers and fluorophore-conjugated antibodies used in the flow cytometry assay, Table S2: Panel of detected single-nucleotide polymorphisms (SNPs), located in the 3′ Regulatory Region 1 (3′RR1) of the human immunoglobulin heavy chain (IgH) locus.
