# Supplementary material for: Associations between Allelic Variants of the Human IgH 3′ Regulatory Region 1 and the Immune Response to BNT162b2 mRNA Vaccine
Source: Vaccines (Basel). 2021 Oct 19;9(10):1207. doi: 10.3390/vaccines9101207 (PMC8540755; doi:10.3390/vaccines9101207)
Supplement: Supplementary file 1 [file vaccines-09-01207-s001.zip › vaccines-1410191-supplementary.pdf]

Associations between allelic variants of the human IgH 3' Regulatory Region 1 and the immune response to BNT162b2 mRNA vaccine

Mattia Colucci, Elisabetta De Santis, Beatrice Totti1, Mattia Miroballo, Francesco Tamiro, Giovanni Rossi, Ada Piepoli, Gabriella De Vincentis, Antonio Greco, Alessandra Mangia, Rossella Cianci, Lazzaro Di Mauro, Giuseppe Miscio & Vincenzo Giambra

Supplementary Information

including

Supplementary Figures S1 and S2

Supplementary Tables S1 and S2

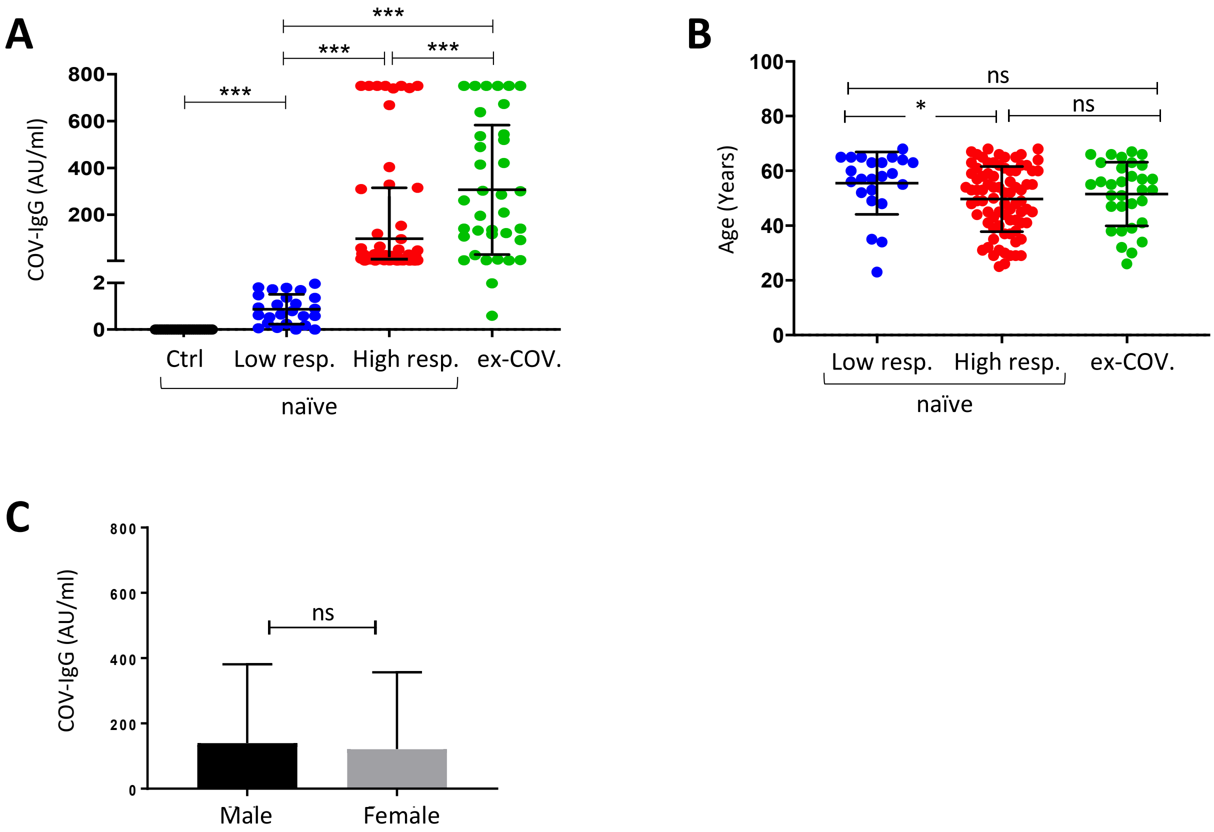

Supplementary Figure S1. anti-SARS-CoV-2 IgG level induced at day 20 by BNT162b2 vaccine. **A**) The anti-SARS-CoV-2 IgG serological level was determined by the SARS-CoV-2 IgG ADVIA Centaur® immunological assay (Siemens Healthineers) and expressed as antibody units per milliliter (AU/mL). Former COVID-19 patients are indicated as "ex-COV". Seronegative participants at day 0 are reported as "naïve" and subdivided in low (N=30) and high (N=93) responsive individuals based on the lower 10th percentile of anti-SARS-CoV-2 IgG sampling distribution which set up the cut-off point at 3.05 AU/ml. The antibody level of 57 not vaccinated healthy donors was also tested and reported in the graph as control (Ctrl). **B**) Age distribution of participants grouped in the considered cohorts, low and high responsive naïve subjects and former ex-COV individuals. **C**) Expression level of anti-SARS-CoV-2 IgG in male (N=77) and female (N=83) voluntaries in response to a single dose of BNT162b2 vaccine at day 20. *ns, not significant; *, p<0.05; ***, p<0.001, **** p<0.0001 (two-tailed unpaired Welch's t-test).*

A

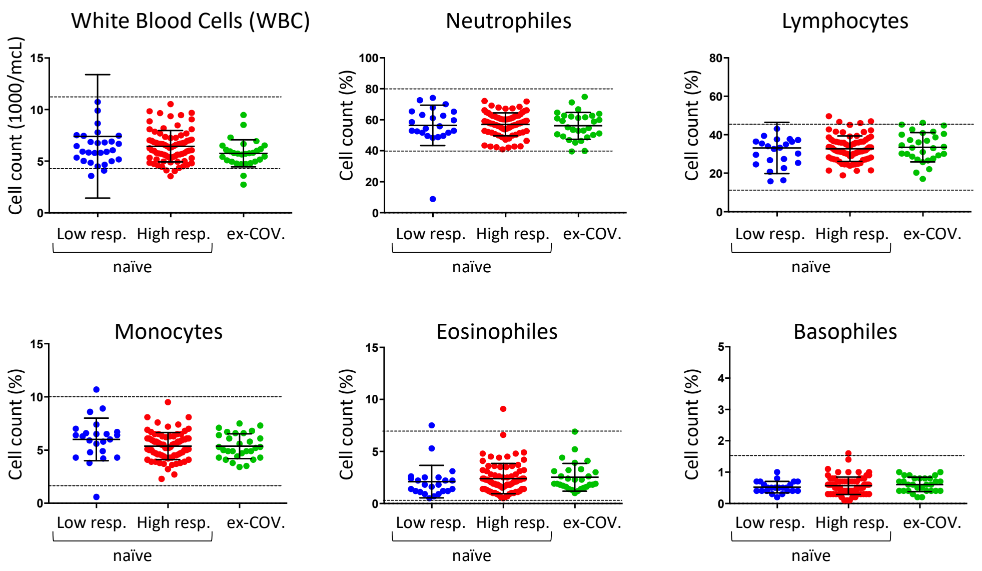

B

	p-value		
**Cell Type**	**Low vs High**	**Low vs ex-COV.**	**High vs ex-COV.**
**White Blood Cells**	0.366	0.366	**0.019**
**Neutrophiles**	0.806	0.959	0.675
**Lymphocytes**	0.900	0.943	0.749
**Monocytes**	0.089	0.123	0.946
**Eosinophiles**	0.269	0.187	0.660
**Basophiles**	0.306	0.149	0.360

Supplementary Figure S2. Distribution of blood cell counts in response to BNT162b2 vaccine at day 20. A) Complete blood counts (CBC) of all participants have been performed at day 20 after exposure at a single dose of BNT162b2 vaccine by the Sysmex XT-4000i automated hematology analyzer. Cell counts are reported as 1000 cells/mcL for white blood cells and as percentage for the other cell types, including neutrophiles, lymphocytes, monocytes, eosinophiles and basophiles. In each graph, the participants are subdivided in naïve low (in blue, N=30), naïve high (in red, N=93) and ex-COV (in green, N=37) responsive groups. The dotted lines specify the standard range of indicated cell counts. **B**) The table shows p-values determined by the two-tailed unpaired Welch's t-test for each comparison reported in (A).

Supplementary Table S1. Panel of cell surface markers and fluorophore-conjugated antibodies used in the flow cytometry assay. APC, allophycocyanine; Cy, cyanine; GFP, green fluorescent protein; PE, phycoerythrin; SB, super bright; RBD, receptor binding domain.

Laser	Detector	Fluorophore	Marker	Antibody Clone	Company	Catalog #
405	448/59	SB436	CD3	SK7	eBioscience™	62-0036-42
	710/45	SB702	CD4	SK3	eBioscience™	67-0047-42
	755 LP	SB780	CD8	RPA-T8	eBioscience™	78-0088-42
488	513/26	GFP	SARS-COV-2-S-RBD			
561	579/16	PE	CD19	SJ25C1	eBioscience™	12-0198-42
	614/20	PE-eFluor 610	CD56	CMSSB	eBioscience™	61-0567-42
	692/75	PE-Cy5	CD38	HIT2	eBioscience™	15-0389-42
640	671/30	APC	CD127	eBioRDR5	eBioscience™	17-1278-42
	722/44	DRAQ7	DNA (viability)			
	795/70	APC-eFluor780	CD25	BC96	eBioscience™	47-0259-42

Supplementary Table S2. Panel of detected single-nucleotide polymorphisms (SNPs), located in the 3' Regulatory Region 1 (3'RR1) of the human immunoglobulin heavy chain (IgH) locus. In the table it is reported the code, genomic coordinates and allelic variant for each SNP. The nested PCRs used for the amplification of different polymorphic regions (see Methods) are also indicated.

SNP code	Genomic Coordinates	Alleles	Nested PCR
rs373084296	chr14:105696525	A>A/G	PCR-1
rs7494440	chr14:105700138	C>A/C/G/T	PCR-2
rs7494441	chr14:105700157	C>C/T	PCR-2
rs61986170	chr14:105700281	G>A/G	PCR-2
rs61986171	chr14:105700316	C>C/G/T	PCR-2
rs12896746	chr14:105700902	A>A/G	PCR-3
rs12896897	chr14:105700958	C>C/T	PCR-3
rs7144089	chr14:105701324	G>A/C/G	PCR-4
rs7143677	chr14:105701369	A>A/G	PCR-4
